# Targeting TNBC: core–shell polycationic polyurea dendrimers with inherent anticancer activity

**DOI:** 10.1002/2211-5463.70144

**Published:** 2026-03-16

**Authors:** Adriana Cruz, Bruna Abreu, Cindy Mendes, Catarina Freitas‐Dias, Filipe Gonçalves, Fernanda Silva, José Ramalho, Saudade André, Vasco D. B. Bonifácio, Jacinta Serpa

**Affiliations:** ^1^ iBB‐Institute for Bioengineering and Biosciences, and Associate Laboratory i4HB‐Institute for Health and Bioeconomy Instituto Superior Técnico Lisbon Portugal; ^2^ iNOVA4Health, NOVA Medical School Faculdade de Ciências Médicas Universidade NOVA de Lisboa Lisbon Portugal; ^3^ Instituto Português de Oncologia de Lisboa Francisco Gentil (IPOLFG), Rua Prof Lima Basto Lisbon 1099‐023 Portugal; ^4^ Bioengineering Department Instituto Superior Técnico, Instituto Superior Técnico Lisbon Portugal

**Keywords:** breast carcinoma, membrane‐targeted therapies, polycationic PURE dendrimers, triple‐negative breast carcinoma

## Abstract

Breast carcinoma (BC) is the most common malignancy in women, with triple‐negative breast cancer (TNBC) making up 10–20% of cases. TNBC has limited targeted therapies and poor survival due to late diagnosis and metastasis. Dendrimers are precise nanostructures with a three‐dimensional globular architecture designed to target the negatively charged membranes of cancer cells. This study evaluated the anticancer potential of two novel core–shell polycationic polyurea (PURE) dendrimers, PURE_G4_‐OEI_48_ and PURE_G4_‐OCEI_24_, targeting BC cell membranes. Both dendrimers selectively interacted with TNBC cells, inducing apoptosis, necroptosis, and ferroptosis. *In vivo*, they reduced tumor volume in HCC1806 xenografts, with PURE_G4_‐OEI_48_ showing no toxicity, while PURE_G4_‐OCEI_24_ induced mild hepatic toxicity. These results suggest PURE dendrimers are promising TNBC treatments, with further modifications needed to enhance efficacy and reduce toxicity.

## Graphical abstract

Core–shell polycationic PURE dendrimers were tested in TNBC‐derived tumor models. Both formulations selectively targeted TNBC and effectively reduced tumor volume. PURE_G4_‐OEI_48_ suppressed tumor growth without detectable toxicity, whereas PURE_G4_‐OCEI_24_, despite showing efficacy, induced hepatic toxicity. Overall, PURE_G4_‐OEI_48_ stands out as a safer and more promising therapeutic approach for TNBC.

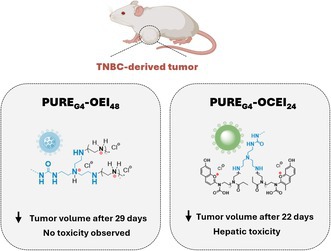

**Abbreviations**
AST: aspartate aminotransferase
BC: breast carcinoma
BSA: bovine serum albumin
cDNA: complementary deoxyribonucleic acid
CTCF: corrected total cell fluorescence
DMEM: Dulbecco's Modified Eagle Medium
EC50: half‐effective concentration
ELISA: Enzyme‐linked Immunosorbent Assays
FBS: fetal bovine serum
FISH: fluorescence *in situ* hybridization
HE: hematoxylin and eosin
LD: lipid droplet
NMR: nuclear magnetic resonance
OCT: optimal cutting temperature
PBS: phosphate‐buffered saline
PE: phosphatidylethanolamine
PFA: paraformaldehyde
PI: propidium iodide
PS: phosphatidylserine
PURE: polyurea
RNA: ribonucleic acid
RT‐qPCR: quantitative reverse transcription polymerase chain reaction
TNBC: triple‐negative breast carcinoma

Breast carcinoma (BC) is the most prevalent malignancy among women globally, ranking first in incidence among women and second overall [1]. Despite advances in treatment, it remains the leading cause of cancer‐related deaths in certain regions, highlighting its persistent status as a global health issue [1, 2]. Factors such as population growth, increased life expectancy, and stressful lifestyles have contributed to rising diagnosis rates, particularly in high‐income countries [3]. BC is broadly divided into five molecular types: normal‐like, luminal A and B, HER2‐enriched, and TNBC. TNBC is a heterogeneous subtype of BC, typically high‐grade, aggressive, and highly proliferative. It does not express hormone receptors and shows no overexpression or amplification of HER2 [4]. These carcinomas account for 10–20% of incident BC cases and have limited targeted therapy options [5]. As anticipated, survival rates for patients diagnosed with TNBC of no special type are low due to frequent late‐stage diagnosis and insufficient therapeutic strategies [6]. In these patients, the risk of early distant recurrence within 5 years of diagnosis is three times higher than in non‐TNBC of no special type patients. The average survival time is around 18 months, with the disease often metastasizing to the central nervous system, liver, lungs, and bones [7, 8]. Anthracycline‐based drugs and taxane chemotherapy remain the gold standard therapeutic strategy for most TNBC patients with a poor prognosis and survival rate [9]. Therefore, new therapeutic approaches are required for the treatment of advanced TNBC disease.

Nanotechnology has emerged over the past decades to improve therapeutics, not only in cancer but also in many other medical conditions. The development of nanoparticles was an important step to improve diagnostic and therapeutic approaches. In cancer therapy, nanocarriers are widely used through various strategies, such as functionalization, simple drug loading, or drug conjugation, showing promising results [10, 11]. Dendrimers are precise nanostructures with a three‐dimensional globular architecture, displaying high mono‐dispersity and multivalency [12, 13]. PURE dendrimers, in particular, have now been reported as an efficient delivery system for ovarian, breast, and lung cancer [14, 15, 16]. Malignant cells reorganize their entire machinery to proliferate and survive over the nonmalignant. These changes directly impact the plasma membrane composition by adding negatively charged biomolecules, such as phosphatidylserine (PS), phosphatidylethanolamine (PE), heparan sulfate, *O*‐glycosylated mucins, or sialylated gangliosides [17, 18, 19, 20]. Given that several studies have reported different classes of dendrimers designed to target the negatively charged membranes of cancer cells [21, 22, 23], PURE dendrimers are envisaged as a powerful alternative.

In this study, we aim to explore the intrinsic anticancer potential of sustainable core–shell polycationic PURE dendrimers, specifically designed to target negatively charged BC cell membranes. Additionally, we seek to uncover the cell death mechanisms triggered by these dendrimers.

## Materials and methods

### Preparation of core–shell polycationic PURE dendrimers

**Fig. 1:**
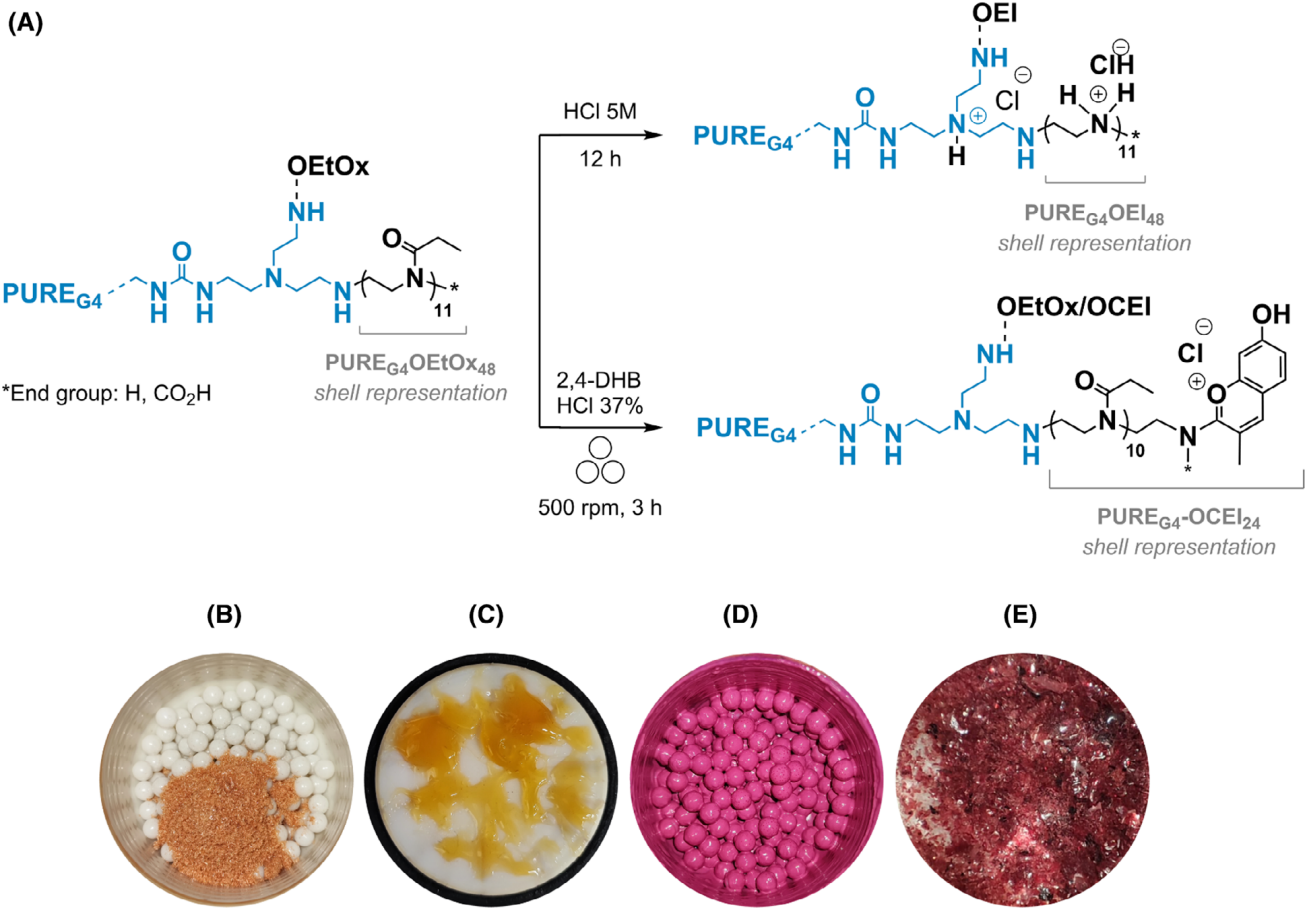
Synthesis of polycationic core–shell PURE dendrimers. (A) PURE_G4_‐OEI_48_ is obtained by full hydrolysis of the *N*‐acetyl groups of PURE_G4_‐OEtOx dendrimer intermediate. PURE_G4_‐OCEI_24_ is obtained via mechanosynthesis by the reaction of the *N*‐acetyl groups of the PURE_G4_‐OEtOx_48_ dendrimer intermediate with 2,4‐dihydroxybenzaldehyde, using HCl as a catalyst, leading to the formation of pending chromylium chloride groups. Only 24 oligo‐oxazolines (50%) from the PURE_G4_ dendrimer shell display one chromylium chloride group in the backbone. The other 24 oligo‐oxazolines remain unchanged. (B) Picture of the zirconium oxide reactor and respective balls (top view) before the reaction, showing the 2,4‐DHB reagent (aldehyde). (C) Zirconium oxide reactor lid before the reaction. The PURE_G4_‐OEtOx_48_ dendrimer intermediate (sticky polymer) was weighed directly in the reactor lid for convenience. (D) Zirconium oxide reactor and respective balls after the reaction (top view). (E) PURE_G4_‐OCEI_24_ crystals after dialysis purification.

The synthesis of the fourth generation polyurea dendrimer (PURE_G4_; Fig. 1) was performed following our previous protocols [24, 25]. The POXylated intermediate PURE_G4_‐OEtOx_48_, a PURE_G4_ dendrimer (core) surface conjugated with oligo‐oxazolines (shell), was first prepared as previously described [26]. This core–shell intermediate dendrimer was used to prepare the final polycationic core–shell dendrimers: a PURE_G4_ dendrimer with an oligoethyleneimine surface (PURE_G4_‐OEI_48_), using our reported protocol [27], and a PURE_G4_ dendrimer with an oligochromyliumethyleneimine surface (PURE_G4_‐OCEI_24_), prepared by a mechanosynthesis protocol (Fig. 1). This protocol was adapted from our previous work, where a polycationic oligo(ethyleneimine‐*N*‐chromylium salt) was prepared by reacting oligo(2‐ethyl‐2‐oxazoline) (OEtOx, the PURE_G4_‐OEtOx_48_ shell) with 2,4‐dihydroxybenzaldehyde (2,4–DHB) and BF_3_.OEt_2_ using a conventional protocol [28].

Briefly, PURE_G4_‐OEtOx_48_ (1.00 g, 0.0111 mmol), 2,4‐DHB (1.08 g, 7.84 mmol), and HCl 37% (1.38 mL) were added to a 50 mL zirconium oxide reactor containing 150 zirconium oxide balls. The mixture was ground for 3 h at 500 ***g*** with rotation inversion cycles of 30 min (5‐s pause between inversion cycles). After this period, 10 mL of methanol were added to the reactor. The methanolic solution was diluted with water and dialyzed for 24 h using a *SnakeSkin*™ dialysis tubing (3.5 K MWCO). The solid that precipitated inside the dialysis tube was filtered and recrystallized from methanol/diethyl ether. A red wine colored solid (0.67 g) was obtained in 95% yield. The molecular weight of PURE_G4_‐OCEI_24_ was calculated by NMR, and only one chromylium chloride group per oligo‐oxazoline chain was found to be formed, in only 24 oligo‐oxazoline chains: *M*_w_ = 63517.4 g·mol^−1^. ^1^H NMR (300 MHz, DMSO‐*d6*) δ (ppm): 8.62–7.94 (m, 96 H, ArH chromylium chloride, 24 chromylium chloride groups from the reaction of the *N*‐acetyl units), 3.64 (m, PURE_G4_ core, partially overlapped with the water signal), 2.27 (1116 H, ArCH_3_ chromylium chloride from 24 groups + NCO*CH*_*2*_CH_3_ from 522 *N*‐acetyl groups), 0.94 (1566 H, NCOCH_2_ *CH*_*3*_ from 522 unreacted methyl groups from the *N*‐acetyl units), as presented in the ^1^H‐NMR spectra (Supplementary Fig. S1). PURE_G4_‐OEI_48_ has a mean diameter of 26.3 ± 2.8 nm with a zeta potential of +46.9 ± 7.9, while PURE_G4_‐OCEI_24_ has a mean diameter of 24.2 ± 0.2 nm and a zeta potential of +25.9 ± 6.2 (Table 1).

**Table 1:** Nanoparticles (PURE_G4_‐OEI_48_ and PURE_G4_‐OCEI_24_) size and zeta potential.

	Size (nm)	Zeta potential
PURE_G4_‐OEI_48_	26.3 ± 2.8	+46.9 ± 7.9
PURE_G4_‐OCEI_24_	24.2 ± 0.2	+25.9 ± 6.2

### Cell culture

Four BC cell lines corresponding to different molecular subtypes were used. Human luminal A, MCF‐7 (RRID: CVCL_0031) (HTB‐22^TM^, ATCC), luminal B, BT‐474 (RRID: CVCL_0179) (HTB‐20^TM^, ATCC), and TNBC, MDA‐MB‐231 (RRID: CVCL_0062) (HTB‐26^TM^, ATCC) and HCC1806 (RRID: CVCL_1258) (CRL‐2335^TM^, ATCC) were obtained from the American Type Culture Collection (ATCC, Manassas, VA, USA). The selected cell lines represent the most common BC molecular subtype, luminal BC (MCF7 and BT474), as well as the most heterogeneous and aggressive subtype TNBC, for which no targeted therapy is currently available (MDA‐MB‐231 and HCC1806). Noncancer cells used as a control included the tubular renal cell line HK2 (RRID: CVCL_YE28) (CRL‐2190^TM^, ATCC), and the keratinocyte cell line HaCaT (RRID: CVCL_0038) (PCS‐200‐011^TM^, ATCC). MCF‐7, BT‐474, MDA‐MB‐231, HK‐2, and HaCaT were cultured in Dulbecco's Modified Eagles Medium 1 (DMEM; 41 965–039, Gibco, Life Technologies), while HCC1806 was cultured in Roswell Park Memorial Institute 1640 (RPMI, BE12‐167F, Lonza). Both media were supplemented with 10% fetal bovine serum (FBS; S 0615, Merck), 1% Antibiotic‐Antimycotic (AA; P06‐07300, PAN Biotech), and 50 μg·mL^−1^ Gentamicin (15750–060, Gibco, Life Technologies). RPMI 1640 was supplemented with 2% L‐glutamine (25 030 081, Gibco, Thermo Fisher Scientific). Cells were maintained at 37 °C in a humidified atmosphere with 5% CO_2_. When confluent, cells were detached with 0.05% Trypsin–EDTA 1× (25300–054, Invitrogen, Thermo Fisher Scientific), for 5 min at room temperature. The cell lines were authenticated by fluorescence *in situ* hybridization (FISH) in the past 3 years. The experiments were performed with mycoplasma‐free cell lines.

### Zeta potential measurements

Cells (1 × 10^6^ cells per flask) were seeded in a T‐25 flask to achieve 80% confluence within 24 h. Afterward, the supernatant was discarded, and the adherent cells were washed and harvested using 0.05% Trypsin–EDTA. The detached cells were centrifuged (255 × *g* for 5 min) and washed with 1 × PBS. Before analysis with the Zetasizer Nano ZS, the cells were centrifuged again (255 × ***g*** for 3 min) and resuspended in ultrapure water. Dynamic light scattering measurements were performed using the Zetasizer Nano ZSP (Malvern Instruments Ltd, UK) in a Folded Capillary Zeta Cell (DTS1070, Malvern Instruments Ltd, UK) at 25 °C.

### Cell proliferation assay

For cell proliferation analysis, cells were seeded in 24‐well plates (5 × 10^4^ cells per well) in complete DMEM. The cells were synchronized under starvation and maintained under control conditions. Cells in suspension in the culture medium and adherent/trypsinized cells were counted at different time points (0, 6, 12, 24, 32, and 48 h) and contrasted by staining with Trypan Blue Stain 0.4% (15 250 061, Gibco™, Thermo Fisher Scientific), using a Bürker cell counting chamber.

### Wound healing assay

The wound healing assay was used to measure 2D directional cell migration, mimicking cell migration during wound healing *in vivo*. Cells were seeded in 12‐well plates (2 × 10^5^ cells per well) with complete DMEM and maintained until reaching 70–80% confluency. Cell proliferation was inhibited with Mitomycin‐C (5 μg·mL^−1^; M4287; Sigma), 3 h before the start of the experiment. In each cell monolayer, a scratch was made along the diameter of the well, and cells in suspension were removed. The wound healing process was monitored by capturing phase‐contrast images (× 200 field) at the following timepoints: 0, 6, 10, 24, 32, and 48 h, using an Olympus IX53 Inverted Microscope. The images were acquired and processed with the Olympus *cellSens* software (https://www.olympus‐lifescience.com/pt/software/cellsens) and analyzed and quantified using the *ImageJ* software.

### Cell death analysis by flow cytometry

Cells were seeded at a density of 1 × 10^5^ cells per well in 24‐well plates and cultured overnight under control conditions. The cells were then exposed to both core–shell polycationic dendrimers PURE_G4_‐OEI_48_ and PURE_G4_‐OCEI_24_ (0.38–51.2 μm) for 24 h. After the experimental conditions, cells in suspension in the conditioned media (supernatants) were collected, and adherent cells were detached with 0.05% Trypsin–EDTA. The cells in the supernatant and detached cells were harvested together by centrifugation, 255 × ***g*** for 2 min. Cells were stained with 0.5 μL Annexin‐V fluorescein (FITC) (640 906, BioLegend, San Diego, CA, USA), in 1 × Annexin‐V binding buffer (10 mm HEPES—pH 7.4, 150 mm NaCl, 2.5 mm CaCl_2_, prepared in 1 × PBS—pH 7.4), and incubated at room temperature, in the dark, for 15 min. Samples were then resuspended in 200 μL in PBS 0.1% BSA and centrifuged at 255 × ***g*** for 2 min. The cells were resuspended in 200 μL of Annexin‐V binding buffer 1 × and 1.25 μL of propidium iodide (PI, 50 μg·mL^−1^; P4170, Sigma‐Aldrich; Darmstadt, Germany) was added 5 min before sample acquisition. Samples were acquired in a BD Accuri C6 Plus Flow Cytometer (Becton, Dickson and Company, Franklin Lakes, NJ, USA) and data analyzed using the *BD CSampler* software. Results were used to define the half‐effect concentration (EC_50_) for both compounds in all BC cell lines.

### Analysis of ferroptosis, apoptosis and necroptosis by flow cytometry

Cells were seeded in a 6‐well plate at 5 × 10^5^ cells per well and kept overnight in control conditions. Cells were then exposed to control, EC_50_ concentrations of PURE_G4_‐OEI_48_ and PURE_G4_‐OCEI_24_, 10% DMSO, and TNFα 50 ng·mL^−1^ conditions for 24 h. BC cells were harvested for simultaneous measurement of reactive oxygen species (ROS), lipid peroxides, cleaved caspase‐3 and cleaved caspase‐9, and p‐RIP3 levels. Cells in suspension in the conditioned media (supernatants) were collected and combined with trypsinized cells and further analyzed for all parameters separately.

For intracellular ROS quantification, cells were centrifuged (1200 × ***g***, 3 min, room temperature), and the supernatant was removed until only 100 μL was left. Then, cells were incubated with the DCF‐DA probe (2 μM, D6883, Sigma‐Aldrich) for 15 min at 37 °C. Afterward, cells were resuspended in 100 μL of DMEM. For lipid peroxide quantification, cells were centrifuged (1200 × ***g***, 3 min, room temperature) and rinsed with PBS 1×. Cell pellets were then resuspended and incubated with BODIPY™ 581/591 C11 in 2% FBS 1 × PBS (2 μm, D3861, Invitrogen) for 30 min at 37 °C in the dark. The excess dye was removed, and cell pellets were resuspended in 2% FBS 1 × PBS.

For cleaved caspase‐3 and cleaved caspase‐9, and p‐RIP3, cells were centrifuged (255 × ***g***, 5 min, 4 °C) and rinsed with 1 × PBS and 0.1% BSA. The pellets were incubated separately with primary antibodies: anti‐cleaved caspase‐3 (96 611, Cell Signaling, Danvers, MA, USA), anti‐cleaved caspase‐9 (9501S, Cell Signaling, Danvers, MA, USA), and anti‐pRIP3 (PA5‐105246, Invitrogen, Waltham, MA, USA) (1 : 500) in 1 × PBS–0.5% BSA–0.1% saponin for 1 h on ice. Cells were rinsed with 1 × PBS and 0.1% BSA (1200 × ***g***, 5 min, 4 °C) and then incubated with secondary anti‐rabbit Alexa Fluor 488 (A11008; Invitrogen, Waltham, MA, USA) (1 : 1000) under the same conditions for 30 min. Cells were washed twice with 1 × PBS and 0.1% BSA and resuspended in 200 μL of the same solution.

All samples were acquired by flow cytometry (BD Accuri C6 Plus Flow Cytometer, Becton, Dickinson and Company, Franklin Lakes, NJ, USA), and data were analyzed using the *BD Accuri C6 Plus software*. The results were expressed as the percentage of FITC‐positive cells.

### Reverse transcription and quantitative PCR (RT‐qPCR)

Total RNA was extracted from MDA‐MB‐231 and HCC1806 cell lines using the RNeasy Mini Extraction Kit (74 104; Qiagen, Hilden, Germany), and cDNA was synthesized from 0.5 μg RNA by SuperScript II Reverse Transcriptase (18080e44; Invitrogen), both according to the manufacturer's protocol. Cells were seeded in 6‐well plates at 5 × 10^5^ cells/well and exposed to EC_50_ concentrations of both PURE_G4_‐OEI_48_ and PURE_G4_‐OCEI_24_ for 24 h.

Quantitative real‐time PCR (qPCR) was performed using SYBR Green PCR Master Mix (04707516001; Roche), according to the manufacturer's protocol, and the specific primers and genes are presented in Table 2. The HPRT gene was used as the housekeeping gene.

**Table 2:** List of primers used in RT‐qPCR assays.

Gene	Protein	Primers (5′–3′)
GCLC	Glutamate‐cysteine ligase	For: ACCAGGGTGATCCTGTCGTA Rev: ATCCCGTTTCTGTGCGACTT
GPX4	Glutathione peroxidase 4	For: GCAGGAGCCAGGGAGTAAC Rev: CCTTGGGTTGGATCTTCATCC
CBS	Glutathione β‐synthase	For: GAGCTCTTGGCCAAGTGTG3 Rev: GCACGTCCACCTTCTCGG3
CTH	Cystathione γ‐lyase	For: GCAGCCACTGTAACTATTACCC Rev: CTGGTGTAATTGCTGCCTCTAG
HPRT	Hypoxanthine phosphoribosyltransferase 1	For: TGACACTGGCAAAACAATGCA Rev: GGTCGTTTTTCACCAGCAAGCT

### xCT glutamate/cystine antiporter levels analyzed by flow cytometry

Cells were seeded in a 12‐well plate at 2 × 10^5^ cells per well and kept overnight in control conditions. They were then exposed to control EC_50_ concentrations of PURE_G4_‐OEI_48_ and PURE_G4_‐OCEI_24_ for 24 h. Adherent cells were detached with 0.05% Trypsin–EDTA and then incubated with rabbit anti‐human xCT antibody (1:500) (ab37185; Abcam, Cambridge, UK) for 1 h in 1 × PBS–0.5% BSA–0.1% saponin at 4 °C. Cells were rinsed with 1× PBS (255 × ***g***, 5 min, 4 °C) and then incubated with secondary anti‐rabbit Alexa Fluor 488 (A11008; Invitrogen, Waltham, MA, US) (1 : 1000) under the same conditions for 30 min. Cells were washed twice with 1 × PBS and 0.1% BSA and resuspended in 200 μL of the same solution. All samples were acquired by flow cytometry (BD Accuri C6 Plus Flow Cytometer, Becton, Dickinson and Company, Franklin Lakes, NJ, USA), and data were analyzed using the *BD Accuri C6 Plus software*. The results were expressed as the percentage of FITC‐positive cells.

### Athymic nude *Mus musculus* orthotopic xenograft BC models

All the murine model procedures were performed according to the rules of Federation for Animal Science Associations (FELASA), accomplishing the 3Rs through evidence‐based guidelines. The murine model studies were approved by the NMS (Ref: 22.05.ORBEA).

Human endpoints for sacrifice will be established as severe weight loss (> 30%), neurological dysfunction, seizures, or moribund condition. To determine the anti‐BC effects of PURE_G4_‐OEI_48_ and PURE_G4_‐OCEI_24_ *in vivo*, a xenograft orthotopic murine model (athymic *Mus musculus*, Charles River, France) of HCC1806 cells was developed by inoculating 2 × 10^6^ cells per mice in the mammary fat pad, as described by our team [29]. After the detection of tumors in the mammary fat pad (approximately 2 weeks after inoculation), their volume was assessed using the formula: volume = 0.5 × length × width^2^. The mice then began treatment with 93.30 μm PURE_G4_‐OEI_48_ and 58.4 μm PURE_G4_‐OCEI_24_ in 1 × PBS. Control mice received injections of 1 × PBS. Injections were administered locally in the tumor every 3 days for 2 weeks, totaling four applications.

Upon euthanasia, using a CO_2_ chamber, primary tumors were extracted, measured for volume determination (the maximum tumor volume was 420 mm^3^), and then frozen and embedded in optimal cutting temperature (OCT) compound. Tumor sections were analyzed by histochemistry for ROS and lipid peroxides. Heart, liver, and kidneys were collected, formalin‐fixed, and paraffin‐embedded. Tissues were then serially sectioned (3 μm), deparaffinized, and stained with hematoxylin and eosin (H&E). The histological evaluation was conducted using the *QuPath* software (https://qupath.github.io/).

Blood samples were collected and centrifuged at 255 × ***g*** for 30 min to harvest serum for toxicity marker detection.

### Nile red and BODIPY™ 581/591 C11 staining of mice frozen sections through fluorescence microscopy

Tumor sections (4 μm) were stained with Nile red and BODIPY™ 581/591 C11 probe and analyzed by fluorescence microscopy. The tissue was embedded in OCT compound before being sectioned. Tumor‐frozen sections were left at room temperature for analysis and then hydrated with 1 × PBS for 15 min. The tissue was incubated with BODIPY™ 581/591 C11 (2 μm) or Nile Red (1 μL·mL^−1^ in PBS 1×) for 20 min and 5 min, respectively, in the dark. The tumor sections were rinsed with 1 × PBS three times, for 5 min each.

Nuclei were counterstained with DAPI (4′‐6‐diamidino‐2‐phenylindole; H‐1200, Vector Labs) and mixed with VECTASHIELD media to mount the glass slides. The tissue was analyzed by standard fluorescence microscopy at a magnification of 400×, using an Axio Imager Z1 microscope (Zeiss) with the *CytoVision*® software. Subsequently, the images were quantified using the *ImageJ* software.

### xCT, Nrf2 and ATG12 immunofluorescence of mice frozen sections

Tumor sections (4 μm) were analyzed by immunofluorescence for xCT, Nrf2, and ATG12 proteins. The tissue was embedded in OCT compound before being sectioned. Tumor‐frozen sections were left at room temperature for analysis and then hydrated with 1 × PBS for 15 min. After fixation (PFA 2%), sections were incubated with 50 mm ammonium chloride (NH_4_Cl) for 10 min. Blocking was performed with PBS 1× − 0.5% BSA − 0.1% saponin‐PBS (w/v/v) for 30 min and rinsed twice with PBS 1× for 5 min. Tumor sections were then incubated with goat anti‐human ATG12 (1 : 100) (Ab0083; Abcam, Cambridge, UK), rabbit anti‐human Nrf2 (1 : 200) (Ab31163; Abcam, Cambridge, UK), and rabbit anti‐human xCT antibody (1 : 500) (ab37185; Abcam, Cambridge, UK) in PBS 1× − 0.5% BSA − 0.1% saponin‐PBS (w/v/v) overnight at 4 °C. After rinsing with PBS 1× for 5 min twice, tumor sections were incubated with secondary antibodies anti‐goat Alexa Fluor 488 (A‐117078; Invitrogen, Waltham, MA, USA) (1 : 500) and anti‐rabbit Alexa Fluor 488 (A11008; Invitrogen, Waltham, MA, US) (1:1000) for 2 h at room temperature. The slides were mounted in VECTASHIELD media with DAPI (4′‐6‐diamidino‐2‐phenylindole) (Vector Labs). The tissue was analyzed by standard fluorescence microscopy at a magnification of 40×, using an Axio Imager Z1 microscope (Zeiss) with the *CytoVision*® software. Subsequently, the images were quantified using the *ImageJ* software.

### Toxicity markers detection by enzyme‐linked immunosorbent assay (ELISA)

To evaluate liver, hepatic, cardiac, and neural damage in murine models upon euthanasia, aspartate aminotransferase (ab263882; Abcam, Cambridge, United Kingdom), α‐fetoprotein (ab210969; Abcam, Cambridge, United Kingdom), Troponin I (ab285235; Abcam, Cambridge, United Kingdom), Park7 (ab277713; Abcam, Cambridge, United Kingdom), and Creatinine (ab65340; Abcam, Cambridge, United Kingdom) ELISA kits were used. The detection assays were performed by following the manufacturer's instructions for blood serum samples.

### Evaluation of *in vitro* hemolytic activity

The hemolysis was evaluated using the protocol described by Sæbø *et al*. [30]. Peripheral blood samples from healthy donors were collected in EDTA tubes and immediately centrifuged at 1700 × ***g*** for 5 min. The supernatant was removed by aspiration, and the erythrocyte *pellet* was washed three times with PBS. After the last washing step, the supernatant was discarded, and the *pellets* were diluted to a 1% erythrocyte suspension in PBS. The erythrocytes were plated in 96‐well polypropylene plates with conical wells and treated with 50 μL of the conditioned media of HCC1806 cells exposed to control conditions and EC_50_ concentrations of PURE_G4_‐OEI_48_ and PURE_G4_‐OCEI_24_. After incubation at 37 °C for 1 h, 2 h, and 4 h, the plates were centrifuged at 1700 × ***g*** for 5 min. Lastly, 50 μL of the supernatants from each well were transferred into a flat‐bottom 96‐well plate. The absorbance of the supernatants was measured at 405 nm in an iMark Microplate Absorbance Reader (Bio‐Rad). Triton X‐100 was used as a positive control, and PBS as a negative control, in identical volumes as test compounds (50 μL).

### Tissue factor detection by enzyme‐linked ImmunoSorbent assay (ELISA)

Detection of the extracellular concentration of the procoagulant protein Tissue factor was made by enzyme‐linked immunosorbent assays (ELISA). Tissue factor is associated with hypercoagulation in cancer patients [31]. Extracellular TF was detected and quantified using the Human Tissue Factor ELISA kit (ab220653; abcam). Prior to the ELISA assays, the conditioned culture media of HCC1806 cells exposed to control conditions and EC_50_ concentrations of PURE_G4_‐OEI_48_ and PURE_G4_‐OCEI_24_ were concentrated in ultrafiltration centrifuge tubes (UFC8010; Merck, Sigma‐Aldrich) at 3220 × ***g*** for 6 min. After the concentration of samples, the assays were performed following the manufacturer's protocols. The original concentrations of Tissue factor in the tested samples were calculated by extrapolating the results measured to the original sample volume.

### Spectrophotometric determination of platelet aggregation

Platelet‐rich plasma (PRP) was gently obtained by Serviço de Imuno‐Hemoterapia at Instituto Português de Oncologia de Lisboa Francisco Gentil (IPOLFG) (IPOLFG‐Ethical committee UIC‐1349). The platelet suspensions were centrifuged at 2650 × ***g*** for 3 min. The platelets were treated with the conditioned culture media of HCC1806 cells exposed to control conditions, and EC_50_ concentrations of PURE_G4_‐OEI_48_ and PURE_G4_‐OCEI_24_, and plated at a concentration of 5 × 10^5^ platelets per μL. Epinephrine addition was used as a positive control. The absorbance was measured after 5, 15, 30, and 60 min, at 595 nm with gentle shaking in an iMark Microplate Absorbance Reader (1 681 130; Bio‐Rad). The relative aggregation for each experimental condition was calculated using the following formula:

$$\%\text{Aggregation}=\frac{{\mathrm{Abs}}_{\text{sample}}}{{\mathrm{Abs}}_{\mathrm{Pos}\;\mathrm{CTL}}}\times 100$$

In which, Abs_sample_ is the absorbance measured in the samples (platelets in conditioned culture media) and Abs_Pos CTL_ is the absorbance measured in the epinephrine positive control.

### Statistical analysis

Statistical analyses were performed in the graphpad prism 8.0 software (www.graphpad.com). Data are presented as mean ± SD. Multiple comparisons between more than one group were performed using one‐way and two‐way ANOVA with Turkey's test.

Half‐EC_50_ was calculated using the log inhibitor vs response – variable slope (four parameter) method. Assays were performed with at least three biological replicates, each with three technical replicates. Statistical significance was established at *p* < 0.05; *p < 0.05, **p < 0.01, ***p < 0.001, ****p < 0.0001.

## Results

### BC cell lines showed a negative zeta potential

**Fig. 2:**
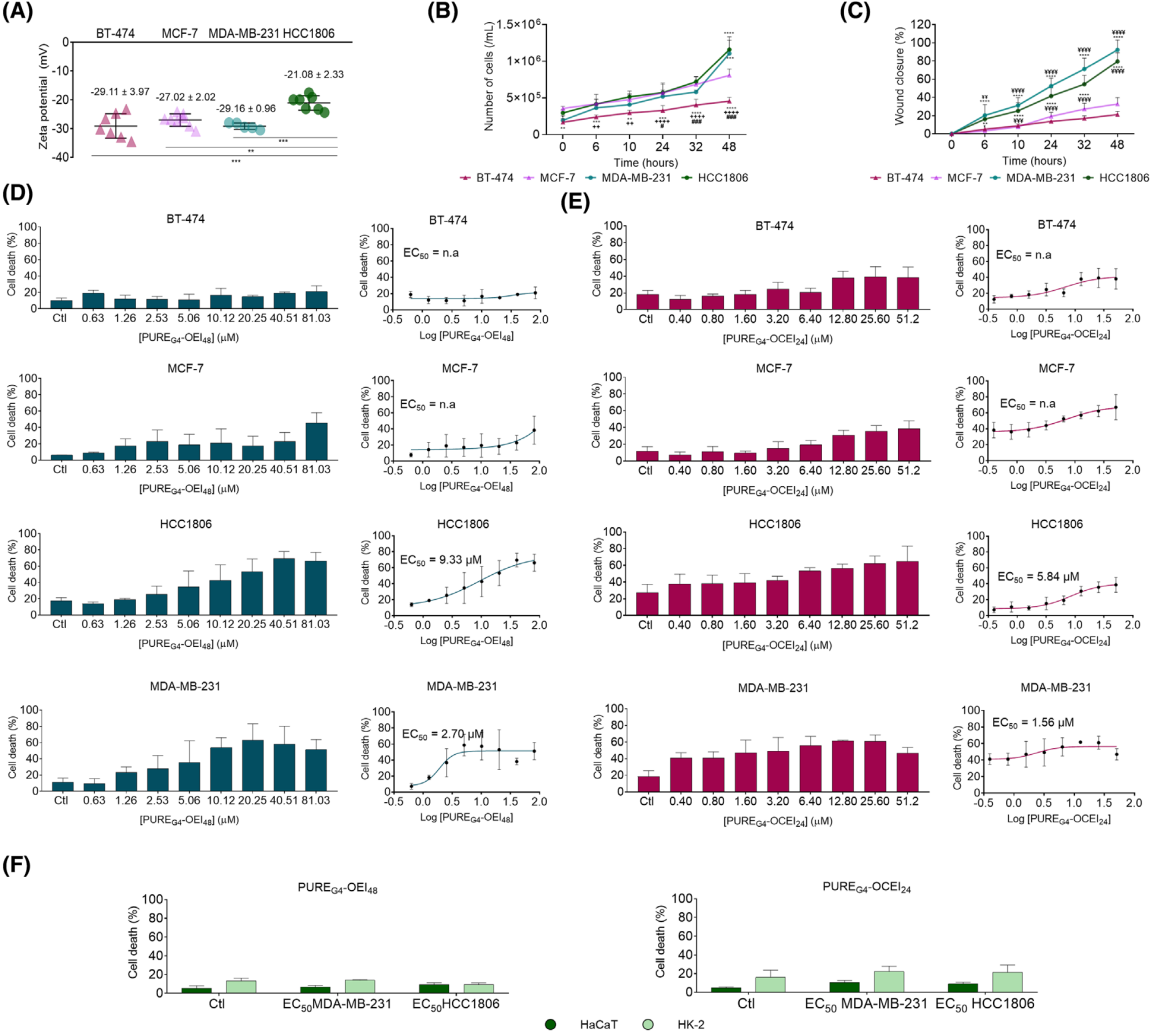
PURE_G4_‐OEI_48_ and PURE_G4_‐OCEI_24_ induce cell death mainly in triple‐negative breast cancer cell lines. (A) The zeta potential (mV) of BC cells (BT‐474, MCF‐7, HCC1806, and MDA‐MB‐231) was recorded in water using the electrophoretic light scattering technique on a Zetasizer Nano ZS analyzer. Statistical significance was determined by one‐way ANOVA with Turkey's multiple comparisons. (B) Cell proliferation curves and (C) wound healing assay were performed in BT‐474, MCF‐7, MDA‐MB‐231, and HCC1806 cell lines under control conditions (two‐way ANOVA, Turkey's test). Cell death was analyzed by flow cytometry using Annexin‐V‐FITC and Propidium iodide labeling in BT‐474, MCF‐7, MDA‐MB‐231, and HCC1806 that were exposed to PURE_G4_‐OEI_48_ (D) and PURE_G4_‐OCEI_24_ (E) for 24 h. (F) Cell death levels in HK‐2 and HaCaT nonmalignant cells exposed to PURE_G4_‐OEI_48_ and PURE_G4_‐OCEI_24_ at EC_50_ values determined for TNBC cells (MDA‐MB‐231 and HCC1806) for 24 h. All experiments were performed in biological triplicates, and data are shown as mean ± SD. (*) Significant difference from MCF‐7, (+) significant difference from HCC1806, (#) significant difference from MDA‐MB‐231, (¥) significant difference from BT‐474, determined by two‐way ANOVA with Turkey's test multiple comparisons.

Zeta potential analysis was conducted to investigate the membrane surface charge properties of BC cells. The four BC cell lines exhibited very similar zeta potential values (Fig. 2A), indicating comparable electrical characteristics between −29.16 and −21.08 mV. Both luminal‐like BC BT‐474 and MCF‐7 cells showed close zeta potential values of −29.11 mV and −27.02 mV, respectively. However, the zeta potential values for TNBC cell lines differed, with MDA‐MB‐231 cells presenting −29.16 mV and HCC1806 cells having the highest zeta potential (−21.08 mV). Because alterations in the cell membrane, caused by cellular processes, such as division and migration, can influence membrane charge [32, 33], the basal proliferation and wound healing capacities of all BC cell lines were assessed. TNBC cell lines were the most proliferative and migratory, with HCC1806 cells showing a higher proliferation rate than MDA‐MB‐231 cells (Fig. 2B), while MDA‐MB‐231 cells were more migratory than HCC1806 (Fig. 2C). BT‐474 was the least proliferative and migratory cell line (Fig 2B,C).

### Core–shell polycationic PURE dendrimers exhibit selective toxicity toward TNBC cell lines

To evaluate the cytotoxic effects of the core–shell polycationic dendrimers PURE_G4_‐OEI_48_ and PURE_G4_‐OCEI_24_, designed to selectively target cancer cell membranes, total cell death and dose‐effect curves were analyzed. Luminal BC cell lines, MCF‐7 and BT474, were less affected by PURE_G4_‐OEI_48_ and PURE_G4_‐OCEI_24_ after 24 h of exposure, showing lower levels of cell death than the TNBC cells (Fig 2D,E). In contrast, TNBC cell lines MDA‐MB‐231 and HCC1806 had higher levels of cell death upon exposure to PURE_G4_‐OEI_48_ and PURE_G4_‐OCEI_24_, exhibiting significantly lower EC_50_ values (Fig 2D,E). Interestingly, TNBC cells did not respond uniformly to the treatments. For both MDA‐MB‐231 and HCC1806, the impact of PURE_G4_‐OEI_48_ (Fig. 2D) started gradually at lower concentrations and intensified with increasing doses, reaching a plateau at 10.12 μm in MDA‐MB‐231 cells. In contrast, both TNBC cell lines were more sensitive to PURE_G4_‐OCEI_24_ at lower concentrations (Fig. 2D). The different sensitivities to the compounds were highlighted by the lower EC_50_ values for PURE_G4_‐OCEI_24_ compared with EC_50_ values for PURE_G4_‐OEI_48_ (Fig 2D,E). Nonmalignant cells, HaCaT and HK‐2, exposed to both dendrimers at the EC_50_ concentrations determined for TNBC cells for 24 h did not show increased cell death (Fig. 2F).

### Apoptotic and necroptotic cell death occurs differently in HCC1806 and MDA‐MB‐231 cells

**Fig. 3:**
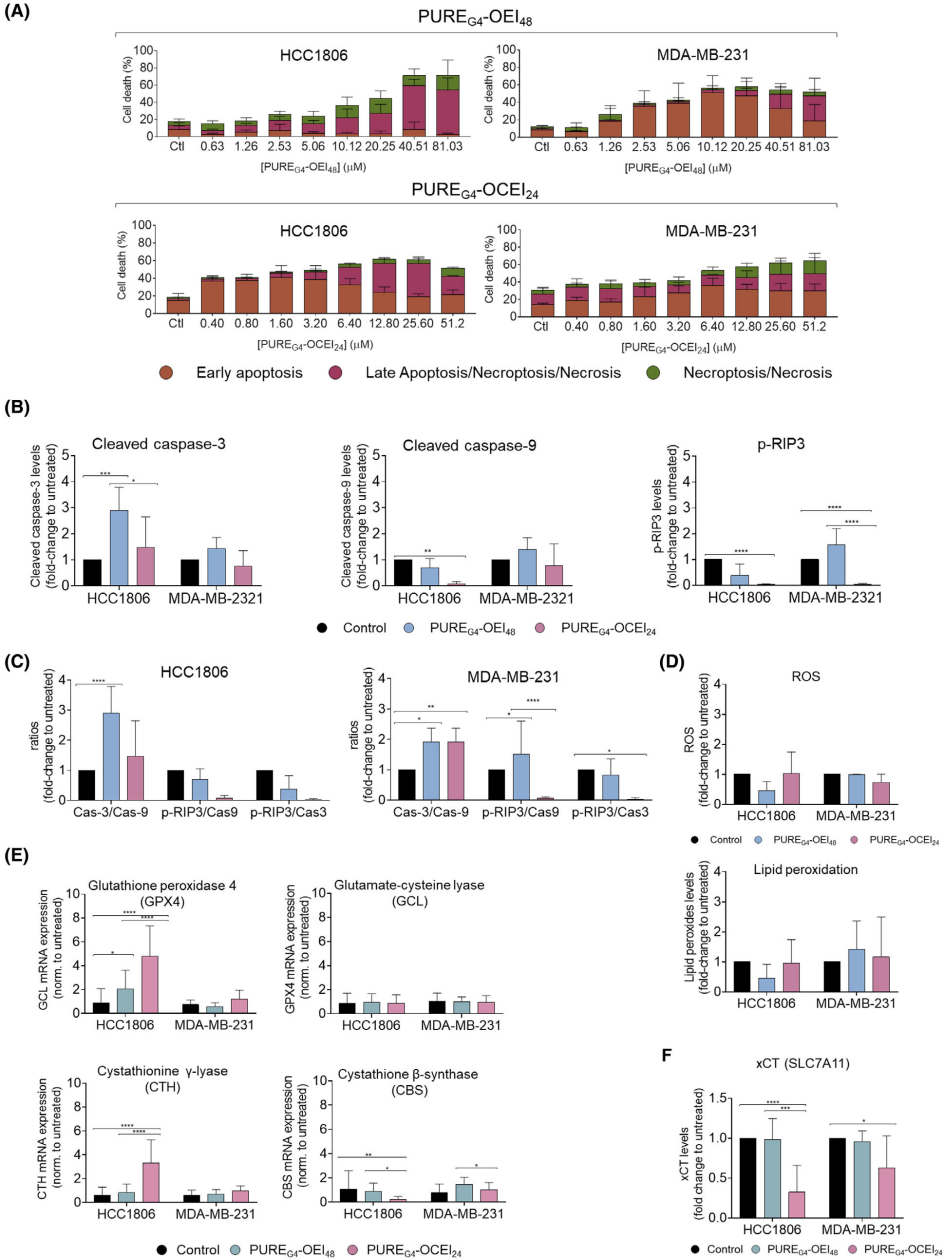
HCC1806 and MDA‐MB‐231 cell lines present different cell death profiles upon PURE_G4_‐OEI_48_ and PURE_G4_‐OCEI_24_ exposure. (A) MDA‐MB‐231 and HCC1806 cells were exposed to PURE_G4_‐OEI_48_ and PURE_G4_‐OCEI_24_; cell death type was characterized based on Annexin‐V and PI labeling, considering that Annexin‐V positive cells are undergoing early apoptosis, cells double‐stained for Annexin‐V and PI are undergoing late apoptosis, necroptosis, or necrosis, and cells positive for PI are undergoing necrosis or necroptosis. (B) HCC1806 and MDA‐MB‐231 cells were exposed to EC_50_ concentrations of PURE_G4_‐OEI_48_ and PURE_G4_‐OCEI_24_ for 24 h, and intrinsic/extrinsic apoptosis and necroptosis were evaluated by flow cytometry considering the levels of cleaved caspase‐3 and cleaved caspase‐9 and p‐RIP3. (C) The predominant cell type occurring in each cell culture condition was addressed by the Cas‐3/Cas‐9, p‐RIP3/cas‐9, and p‐RIP3/cas‐3 ratios. (D) By flow cytometry, cytoplasmic reactive oxygen species (ROS) were measured using staining of the cells with DCF‐DA, and ferroptosis was assessed by BODIPY™ 581/591 C11 labeling to monitor lipid peroxidation. (E) The expression of genes involved in cellular redox control was quantified by qPCR in cells exposed to PURE_G4_‐OEI_48_ and PURE_G4_‐OCEI_24_. (F) The levels of the cystine/glutamate antiporter SLC7A11 (xCT) were assessed by flow cytometry in HCC1806 and MDA‐MB‐231 cells exposed to the EC_50_ concentrations of PURE_G4_‐OEI_48_ and PURE_G4_‐OCEI_24_. All experiments were performed in biological triplicates, and data are shown as mean ± SD. Statistical significance was determined using two‐way ANOVA followed by Sidak's multiple comparison test for panels A and B, and Tukey's multiple comparison test for panels E and F.

Based on the Annexin‐V (Ann) and propidium iodide (PI) staining profiles, we observed that TNBC cells showed different cell death patterns under the same conditions (Fig 3A,B). MDA‐MB‐231 cells exhibited an increase in early apoptosis with PURE_G4_‐OEI_48_ concentrations, but at concentrations above 20.25 μm, a notable shift toward late apoptosis/necroptosis/necrosis was observed (Fig. 3A). This trend was also observed for MDA‐MB‐231 cells exposed to PURE_G4_‐OCEI_24_ (Fig. 3A). For concentrations ranging from 0.40 to 3.20 μm, early apoptotic cell levels increased until reaching the EC_50_ (1.56 μm) at 50%, after which a shift toward late apoptosis/necroptosis/necrosis was observed (Fig. 3A).

In HCC1806 cells, upon treatment with PURE_G4_‐OEI_48_, cells were distributed in early and late apoptosis/necroptosis/necrosis at lower concentrations, reaching 50% cell death at 10.12 μm (EC_50_ = 9.33 μm) (Fig. 3A). Above 10.12 μm, most cells exhibited an apoptotic/necroptotic/necrotic profile. When exposed to PURE_G4_‐OCEI_24_, HCC1806 showed early apoptosis induction in a concentration‐dependent manner between 0.40 and 6.40 μm, with early apoptosis being the prevalent cell death pattern in all tested concentrations (Fig. 3A).

### PURE_G4_ ‐OEI_48_ activated apoptosis in HCC1806 cells and necroptosis in MDA‐MB‐231 cells, while PURE_G4_ ‐OCEI_24_ only activated intrinsic apoptosis in HCC1806

Due to the limitation in distinguishing cells undergoing late apoptosis from those undergoing necroptosis and necrosis using annexin‐V‐FITC and PI by flow cytometry, we analyzed the expression of key players in apoptotic (cleaved caspase‐3 and cleaved caspase‐9) and necroptotic (p‐RIP3) cell death by flow cytometry (Fig 3B,C).

PURE_G4_‐OEI_48_ at EC_50_ concentrations induced distinct effects on cleaved caspase‐3 and cleaved caspase‐9 and p‐RIP3 cell death markers' expressions (Fig. 3B), compared with untreated controls. In HCC1806, cleaved caspase‐3‐positive cells increased in treated cells (2.89‐fold change) while positive cells for cleaved caspase‐9 and p‐RIP3 only showed 0.69‐ and 0.37‐fold change, respectively, upon treatment. In contrast, MDA‐MB‐231 cells had an increment in all cell death markers, showing a fold change to untreated between 1.39 and 1.56 (Fig. 3B). The ratios cleaved caspase‐3/cleaved caspase‐9, p‐RIP3/cleaved caspase‐9, and p‐RIP3/cleaved caspase‐3 were also determined (Fig. 3C). Compared with the untreated control, HCC1806 showed a 1.45‐fold change for cleaved caspase‐3/cleaved caspase‐9. This suggests that in treated cells, extrinsic apoptosis prevails even more over intrinsic apoptosis. Showing a different tendency, the ratios p‐RIP3/cleaved caspase‐9 and p‐RIP3/cleaved caspase‐3, 0.08 and 0.1‐fold change, respectively, indicate that in treated cells, apoptotic cell death generally prevails over necroptosis. MDA‐MB‐231 cells (Fig. 3C) showed a 1.45‐fold change increase in the cleaved caspase‐3/cleaved caspase‐9 ratio compared with untreated cells, suggesting that, again, treated cells activate the extrinsic apoptotic machinery over the intrinsic one. The p‐RIP3/cleaved caspase‐9 was 1.51 (fold change) higher in treated cells, whereas the p‐RIP3/cleaved caspase‐3 ratio was lower than the untreated control (0.8‐fold change). Again, those ratios support that in treated cells, extrinsic apoptosis prevails over necroptosis.

TNBC cells treated with PURE_G4_‐OCEI_24_ (Fig. 3B) showed low levels of apoptosis and necroptosis markers (fold change to untreated), except for HCC1806, which showed a 1.46‐fold change in cleaved caspase‐3. These results are translated into a higher Cas3/Cas9 ratio in HCC1806 (18.61‐fold change to untreated) (Fig. 3C), indicating a preference for the intrinsic apoptotic pathway. Regarding MDA‐MB‐231, it seems that PURE_G4_‐OCEI_24_ did not activate either apoptosis or necroptosis in this cell line (Fig 3B,C). The experimental controls, shown in Fig. S2A,B, included untreated cells, cells treated with 10% DMSO (v/v) to induce apoptosis through cleaved caspase‐3 activation [34], and cells exposed to 50 ng·mL^−1^ of TNFα, which can trigger cleaved caspase‐3 (32) and activate p‐RIP3 [35].

### The levels of ROS and the extent of lipid peroxidation vary in TNBC cells treated with PURE_G4_ ‐OEI_48_ and PURE_G4_ ‐OCEI_25_

Ferroptosis was assessed by measuring intracellular ROS levels through the DCF‐DA probe and lipid peroxidation via incubation with the BODIPY^TM^ 581/591 C11 dye (Fig. 3D). Compared with the untreated control, PURE_G4_‐OEI_48_ tends to reduce ROS levels and lipid peroxidation in HCC1806 cells, while PURE_G4_‐OCEI_24_ shows no significant effect (Fig. 3D). The dichotomy of ROS levels/lipid peroxidation follows the same tendency in HCC1806 cells exposed to both dendrimers, decreasing or increasing depending on the treatment (Fig. 3D). In MDA‐MB‐231 cells, ROS levels were only affected in cells exposed to PURE_G4_‐OCEI_24_, but lipid peroxides tended to increase for PURE_G4_‐OEI_48_ (1.41‐fold change) and PURE_G4_‐OCEI_24_ (1.16‐fold change), compared with the untreated control (Fig. 3D). ROS levels were also measured in cell death controls, namely untreated cells, cells exposed to 10% DMSO (v/v), and cells exposed to 50 ng/mL of TNFα (Suppl. Fig. S2 C).

### Core–shell polycationic PURE dendrimers impact the relative mRNA expression levels of genes involved in redox control, and PURE_G4_ ‐OCEI_25_ decreases xCT levels

Relative mRNA expression levels were assessed by RT‐qPCR in HCC1806 and MDA‐MB‐231 cells exposed to EC_50_ concentrations of both dendrimers (Fig. 3E). In HCC1806, the glutathione peroxidase‐4 (GPX4) encoding gene was not affected by either of the dendrimers (Fig. 3E). Regarding CTH mRNA expression, PURE_G4_‐OCEI_24_ induced increased levels of CTH (3.32‐fold) in HCC1806 compared with the control, while PURE_G4_‐OEI_48_ did not induce alterations (Fig. 3E). The gene encoding the limiting enzyme involved in glutathione biosynthesis (GCL) was significantly increased by both dendrimers in HCC1806 cells (Fig. 3E). Cystathionine β‐synthase (CBS) mRNA expression levels were not affected by the dendrimers in HCC1806 (Fig. 3E). In MDA‐MB‐231, no alterations were observed in the expression of GPX4, CTH, and GCL, while CBS mRNA expression levels were increased by PURE_G4_‐OEI_48_ exposure (Fig. 3E).

The glutamate/cystine antiporter xCT levels were significantly decreased upon exposure to PURE_G4_‐OCEI_24_ in both HCC1806 and MDA‐MB‐231 cell lines (Fig. 3F).

### PURE_G4_ ‐OEI_48_ and PURE_G4_ ‐OCEI_24_ reduced the tumor volume with low systemic toxic impact

**Fig. 4:**
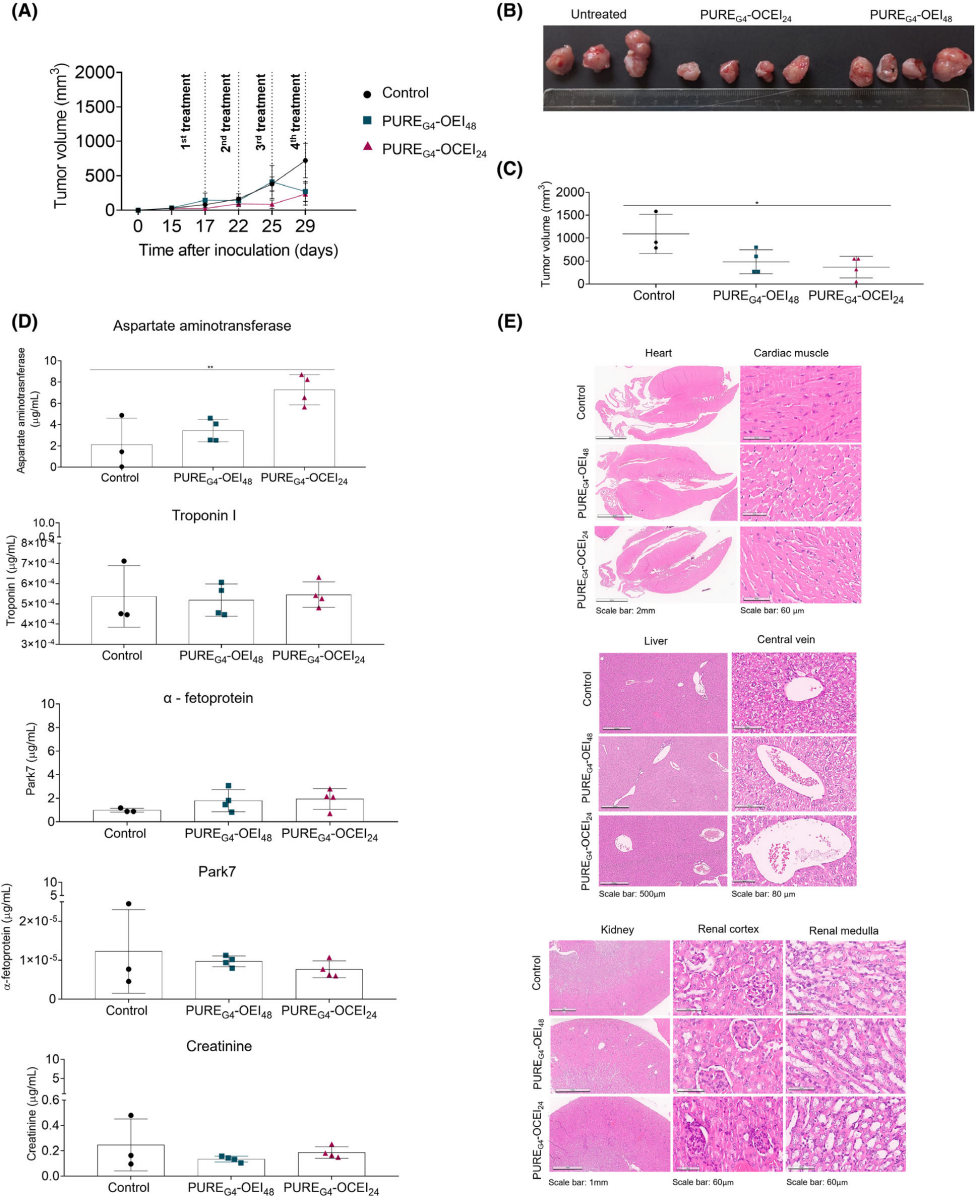
PURE_G4_‐OEI_48_ and PURE_G4_‐OCEI_24_ reduce tumor volume in an *in vivo* BC murine model. HCC1806 cells were inoculated in the mammary fat pad to induce tumors in a xenograft murine model (thymic nude *Mus musculus*). Treatment was administered every 3 days, beginning 17 days postinoculation and continuing until day 29, and (A) a tumor growth curve was constructed by measuring the weight and length and calculating the tumor volume. Upon euthanasia, (B) tumors were collected, and (C) the volume calculated. (D) The levels of toxicity markers were assessed by ELISA in the peripheral blood serum of mice: aspartate aminotransferase and α‐fetoprotein (hepatic toxicity), troponin I (cardiac toxicity), Park7 (neuronal toxicity), and creatinine (liver toxicity). (E) Representative sections of paraffin‐embedded, and hematoxylin and eosin (H&E) stained heart, liver, and kidney were evaluated. Comprehensive areas and magnified details of functional structures are presented for each organ. Images were captured in an imager Aperio AT2 Leica, using the software *Aperio Scanner Console* version 102.0.7.5. All experiments were performed in biological triplicates, and data are shown as mean ± SD. Statistical significance was determined using one‐way ANOVA followed by Sidak's multiple comparison test. Scale bars: heart and kidney, 2 mm; cardiac muscle, renal cortex, and renal medulla, 60 μm; liver, 500 μm; central vein, 80 μm.

To determine the anti‐BC effects of the dendrimers *in vivo*, a xenograft orthotopic murine model was developed by inoculating HCC1806 cells into the mammary fat pad of athymic mice. Tumor volume was measured before the first dose administration, on day 17, and every 3 days during the two‐week treatment period (Fig. 4A). Tumors in mice treated with PURE_G4_‐OEI_48_ grew similarly to untreated tumors, but a decrease in tumor size was observed on day 25, following the third PURE_G4_‐OEI_48_ administration (Fig. 4A). In contrast, tumors in mice treated with PURE_G4_‐OCEI_24_ exhibited slower growth from the first PURE_G4_‐OCEI_24_ administration, compared with the untreated control group of mice (Fig. 4A). Mammary tumors were extracted upon euthanasia and their volume was measured (Fig 4B,C). Both core–shell PURE_G4_‐OEI_48_ and PURE_G4_‐OCEI_24_ presented significant efficacy in reducing the tumor volume (Fig 4B,C).

To evaluate potential systemic toxicity induced by the dendrimers, key toxicity markers, such as aspartate aminotransferase, α‐fetoprotein, troponin I, Park7, and creatinine, were measured in mice peripheral blood serum (Fig. 4D). The levels of aspartate aminotransferase tended to increase in mice treated with PURE_G4_‐OEI_48_ (3.43 μg·mL^−1^), and it was significantly increased in mice treated with PURE_G4_‐OCEI_24_ (7.28 μg·mL^−1^). No significant alterations were observed for α‐fetoprotein, troponin I, Park7, and creatinine in mice treated with PURE_G4_‐OEI_48_ and PURE_G4_‐OCEI_24_ (Fig. 4D).

The histological analysis of H&E‐stained sections revealed no morphological alterations in the liver, heart, and kidney (Fig. 4E), such as inflammation, necrosis, or fibrosis, compatible with no cytotoxic effects driven by PURE_G4_‐OEI_48_ and PURE_G4_‐OCEI_24_ exposure. The absence of histological alterations reinforces that these dendrimers do not induce significant systemic toxicity under the applied conditions.

### PURE_G4_ ‐OEI_48_ and PURE_G4_ ‐OCEI_24_ triggered oxidative stress, while PURE_G4_ ‐OCEI_24_ also induced autophagy in tumors

**Fig. 5:**
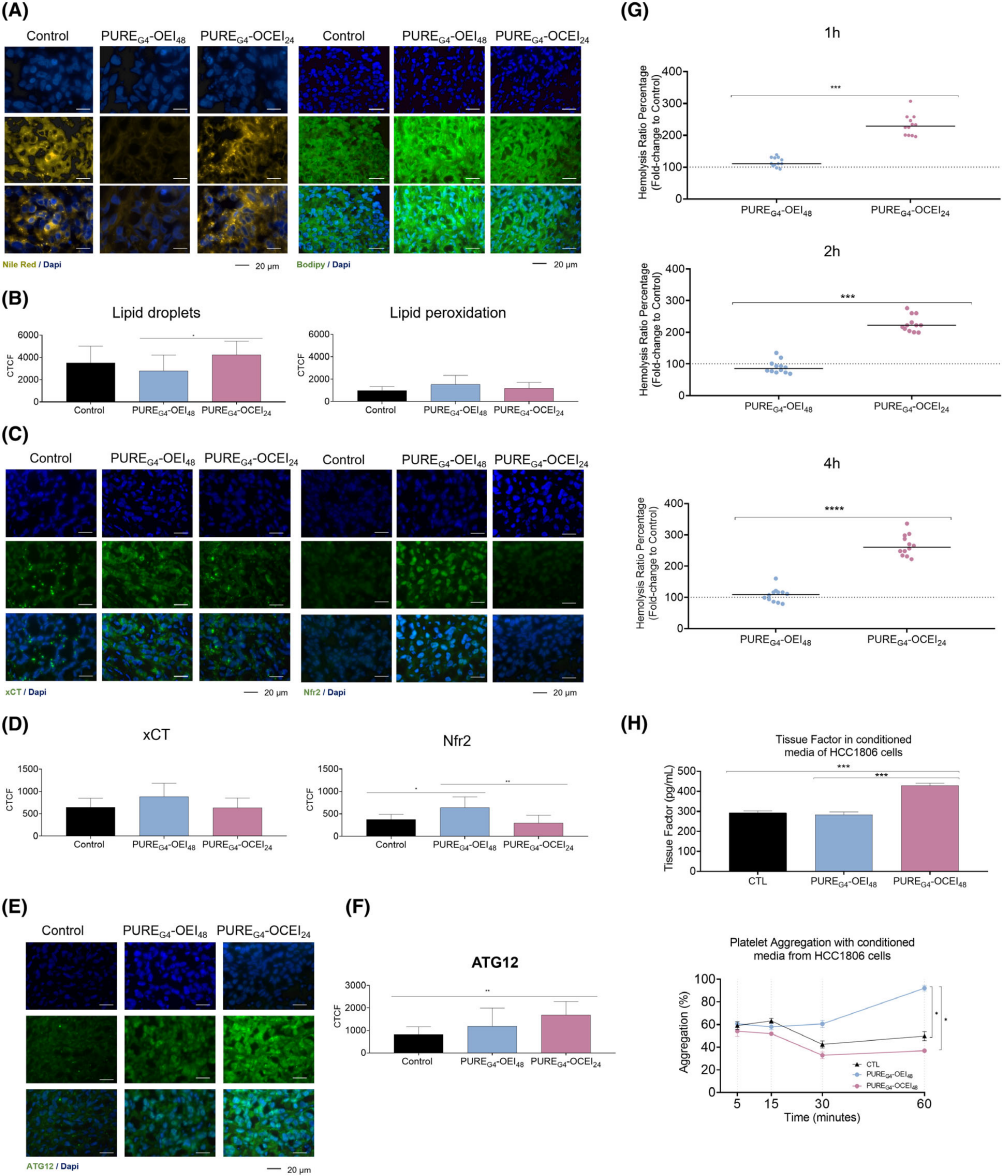
PURE_G4_‐OEI_48_ effectively triggers oxidative stress in mice while PURE_G4_‐OCEI_24_ induces autophagic survival mechanisms. (A) Representative images of fluorescent microscopy for LD content detected with Nile red cytochemistry and lipid peroxides levels detected with BODIPY^TM^ 581/591 C11 in HCC1806‐derived xenograft tumors developed in untreated and PURE_G4_‐OEI_48_‐treated and PURE_G4_‐OCEI_24_‐treated athymic nude mice. (B) Quantification of LD and lipid peroxides levels by corrected total cell fluorescence (CTCF). (C) xCT and Nrf2 expression levels in xenograft tumors calculated through (D) CTCF quantification. ATG12 immunofluorescence (E) in tumors exposed to PURE_G4_‐OEI_48_ and PURE_G4_‐OCEI_24_ and CTCF calculation to determine ATG1 levels in tumor sections (F). Hemolysis was followed for 1, 2, and 4 h by exposing whole blood to conditioned culture media of HCC1806 cells exposed to control conditions, and EC_50_ concentrations of PURE_G4_‐OEI_48_ and PURE_G4_‐OCEI_24_ (G). Tissue factor was quantified in conditioned culture media of HCC1806 cells exposed to control conditions and EC_50_ concentrations of PURE_G4_‐OEI_48_ and PURE_G4_‐OCEI_24_ by ELISA (H). Platelets were exposed to conditioned culture media of HCC1806 cells exposed to control conditions and EC_50_ concentrations of PURE_G4_‐OEI_48_ and PURE_G4_‐OCEI_24_ and aggregation was measured after 5, 15, 30, and 60 min, at 595 nm (I). All experiments were performed in biological triplicates and data are shown as mean ± SD. Statistical significance was determined using one‐way ANOVA followed by Tukey's multiple comparison test for panels D, F, G, and one‐way ANOVA followed by Dunnett's test for panel B, and two‐way ANOVA followed by Tukey's multiple comparison test for panel H. Scale bar: 20 μm.

To assess the redox imbalance induced by PURE_G4_‐OEI_48_ and PURE_G4_‐OCEI_24_, the lipid droplets (LD) content and lipid peroxide levels were evaluated. It was observed that the levels of LD and lipid peroxidation showed an inverse pattern, indicating a dynamic process. Tumors from mice treated with PURE_G4_‐OEI_48_ showed significantly decreased levels of LD compared with tumors from mice treated with PURE_G4_‐OCEI_24_ (Fig 5A,B). Regarding lipid peroxides, tumors from mice treated with PURE_G4_‐OEI_48_ tended to increase lipid peroxidation compared with tumors from mice treated with PURE_G4_‐OCEI_24_ (Fig 5A,B). The levels of LD and lipid peroxides in tumors from mice treated with PURE_G4_‐OCEI_24_ were similar to tumors from untreated mice (Fig 5A,B).

Redox imbalance and treatment response *in vivo* were also evaluated by measuring the levels of xCT transporter and Nrf2, the master regulator of oxidative stress responses, including xCT expression [36, 37]. PURE_G4_‐OEI_48_ indeed promotes an increase in xCT and Nrf2 (Fig 5C,D). The xCT levels for tumors exposed to PURE_G4_‐OCEI_24_ remain unchanged when compared with control (Fig 5C,D), but Nrf2 expression decreased in treated tumors (Fig 5C,D).

ATG12 levels were assessed to explore autophagy activation, as an attempt to rescue cell survival [38, 39]. Cells exposed to PURE_G4_‐OEI_48_ tended to increase the ATG12 levels compared with control, though not in a significant way (Fig 5E,F). Importantly, PURE_G4_‐OCEI_24_ exposure induced a significant increase in ATG12 levels, indicating the activation of autophagy (Fig 5E,F).

### PURE_G4_ ‐OEI_48_ and PURE_G4_ ‐OCEI_24_ impact differently blood coagulation and hemolysis

In order to disclose the putative effects of dendrimers in blood coagulation and hemolysis, we performed platelet aggregation and hemolysis assays together with the quantification of tissue factor in the HCC1806 conditioned media. PURE_G4_‐OEI_48_ did not show alterations in hemolysis or tissue factor production, but it increased platelet aggregation (Fig. 5G–I). PURE_G4_‐OCEI_24_ induced hemolysis and increased tissue factor production, without interfering with platelet aggregation (Fig. 5G–I). Therefore, PURE_G4_‐OEI_48_ offers more safety as an anticancer drug.

## Discussion

TNBC constitutes a heterogeneous group of BC, with an overall poor prognosis. The lack of expression of hormonal receptors and the HER‐2 receptor limits targeted therapy. TNBC exhibits greater cell invasiveness and higher metastatic rates to the liver, bones, and lungs, compared with luminal BC [7]. For these patients, the overall survival rate is about 18 months compared with 5 years for HR and HER2‐positive BC [40]. Chemotherapy remains the gold standard for TNBC patients, and the lack of predictors of chemoresistance makes the design of new therapies an opportunity to improve TNBC treatment [41, 42].

We explored the intrinsic anticancer potential of two novel core–shell polycationic PURE dendrimers, PURE_G4_‐OEI_48_ and PURE_G4_‐OCEI_24_, against BC, and importantly, we reported selectivity for TNBC over luminal BC cell lines (Fig 2D,E). Both dendrimers PURE_G4_‐OEI_48_ and PURE_G4_‐OCEI_24_ display positively charged groups on the surface (Fig. 1A) to specifically target cell membranes that possess an increment of negatively charged groups at the surface. The rationale supporting this cancer cells' targeting relates to both the topical and systemic application of dendrimers. In topical applications—where dendrimers are applied directly to the tumor—they come into contact with both cancerous and noncancerous cells within the tumor microenvironment. However, due to the unique chemical composition and charge of cancer cell membranes, dendrimers preferentially interact with cancer cells over stromal cells. In systemic applications, tumor‐associated blood vessels are typically leaky and unstable [43, 44], allowing dendrimers circulating in the bloodstream to extravasate more readily into the tumor tissue, while being less likely to accumulate in healthy tissues with stable vasculature. In the tumor, the dendrimers will again preferentially interact with cancer cells.

Cancer cells alter their metabolism to support uncontrolled growth, with lipid metabolic remodeling playing a central role. This allows malignant cells to utilize lipids for energy and biosynthesis, as well as reorganize their plasma membrane [45, 46, 47, 48]. Normally, phospholipids are asymmetrically distributed between the outer and inner leaflets of the plasma membrane, with zwitterionic phospholipids, such as phosphatidylcholine (PC) and PE, located on the surface [49, 50, 51]. In cancer, this asymmetry is lost, and negatively charged phospholipids, such as PS and PE, are translocated to the outer leaflet [17, 18, 20]. Metastatic breast cancer cells show increased PS content compared with nonmetastatic cells [52], and their membranes exhibit greater deformability, with lower cholesterol and sphingomyelin [53]. Other components, including mucins, glycosaminoglycans (GAGs), and sialic acid residues, contribute to a negatively charged surface [54, 55]. These changes alter the cell's zeta potential (ζ), reflecting the surface charge, and lead to membrane depolarization (less negative zeta potential) rather than hyperpolarization (more negative zeta potential) [56, 57].

Zeta potential, the electrical potential beyond the surface (few nanometers) of any suspension in water [56], is linked to membrane potential (*V*_*m*_), a voltage difference created by the unequal distribution of ions across the cell membrane [56]. The heterogeneity associated with TNBC [58] was corroborated by zeta potential measurements (Fig. 2A). The HCC1806 TNBC cells were revealed to be more depolarized, having a zeta potential around −21.08 mV, while MDA‐MB‐231 showed a zeta potential of −29.16 mV, following reported data [59]. In contrast, luminal‐like BT‐474 and MCF‐7 cells showed a more similar zeta potential of −29 mV and −27 mV, respectively (Fig. 2A). Michael Hughes [56] suggests that hyperpolarized membrane potential values are accompanied by hyperpolarized zeta potential values [56]. Hypothesizing that depolarized membrane potential is also associated with depolarized zeta potential and recognizing that proliferative cells exhibit a less negative membrane potential [60], our findings can be supported. The high proliferative rates of HCC1806 (Fig. 2B) are aligned with the depolarized zeta potential values observed in these cells (Fig. 2A). MDA‐MB‐231 cells are less proliferative than HCC1806 cells, which may be associated with their more polarized zeta potential (Fig 2A,B). Among luminal‐like BC cells, even with no significant differences, MCF‐7 cells showing depolarized zeta potential presented a higher proliferative rate than BT‐474 (Fig 2A,B). Overall, the TNBC cell lines were the most proliferative and migratory among all BC cell lines tested (Fig 2B,C), which agrees with the more aggressive phenotype attributed to this BC molecular type. The dichotomy between proliferation and migration may explain the shifting dominance of these cells in terms of their proliferative and migratory abilities. Cell proliferation and migration are asynchronous processes [61] that may function in a pulse‐chase manner, yet both are crucial for cancer progression, contributing to some of the most aggressive phenotypes, such as TNBC.

Both PURE_G4_‐OEI_48_ and PURE_G4_‐OCEI_24_ dendrimers contain positively charged groups that selectively interact with negatively charged membranes via electrostatic interactions [62]. Considering that BT‐474 and MDA‐MB‐231 cells showed higher zeta potential values, an increasing specificity of these compounds for these cell lines was expected. But, in terms of cell death, we interestingly observed that TNBC cell lines MDA‐MB‐231 and HCC1806 are more sensitive to core–shell polycationic PURE dendrimers, showing a gradual dose‐dependent effect, particularly for PURE_G4_‐OEI_48_ (Fig. 2D). In contrast, cells treated with PURE_G4_‐OCEI_24_ are immediately affected at lower concentrations. To better understand the cell death profiles induced in TNBC cells after treatment, we considered cells typically classified as early apoptotic (Ann+), necrotic or necroptotic (PI+) [35, 63], and late apoptotic/necrotic/necroptotic (Ann+/PI+) [64]. PURE_G4_‐OEI_48_ primarily induced extrinsic apoptosis, evidenced by increased cleaved caspase‐3 levels and high caspase‐3/caspase‐9 ratios in both cell lines (Fig. 3B,C). In MDA‐MB‐231 cells, necroptosis was also activated by this treatment, as indicated by elevated p‐RIP3 expression (Fig. 3B), suggesting a shift from apoptosis to necroptosis under near‐toxic conditions. This transition from apoptosis to necroptosis may reflect the plasticity of cell death pathways in response to stress. In contrast, PURE_G4_‐OCEI_24_ induced minimal apoptotic or necroptotic activity in MDA‐MB‐231 cells (Fig. 3B,C) but effectively activated extrinsic apoptosis in HCC1806 cells, with significantly reduced cleaved caspase‐9 and p‐RIP3 levels (Fig. 3B). These results highlight the differential sensitivity of TNBC cell lines, with HCC1806 favoring apoptosis over necroptosis for both dendrimers, while MDA‐MB‐231 displayed greater reliance on necroptosis in response to PURE_G4_‐OEI_48_. The distinct cell death profiles emphasize the need for therapies tailored to specific cell types within heterogeneous groups, such as TNBC.

Ferroptosis, a newly discovered regulated iron‐dependent type of cell death distinct from apoptosis, necrosis, and necroptosis [65, 66], was also addressed in this study. Despite the lack of molecular markers strictly specific for ferroptosis, three essential features are established: dysfunction in lipid peroxide scavenging due to the glutathione and cysteine imbalance and GPX4 inhibition; the presence of redox‐active iron; and oxidation of polyunsaturated fatty acid (PUFA)‐containing phospholipids [67, 68]. Therefore, monitoring ROS levels and lipid peroxidation provides insights into the cellular ferroptosis activation state. ROS and lipid peroxide levels were assessed in HCC1806 and MDA‐MB‐231 cells, using DCF‐DA and BODIPY™ 581/591 C11 probes, respectively (Fig. 3D). In HCC1806 cells, PURE_G4_‐OEI_48_ clearly reduced ROS levels, while PURE_G4_‐OCEI_24_ had no effect. Lipid peroxide levels followed the same tendency, suggesting PURE_G4_‐OEI_48_ disturbs the redox balance and ferroptosis was triggered (Fig. 3D). In MDA‐MB‐231 cells, PURE_G4_‐OEI_48_ did not affect ROS levels but increased the content of lipid peroxides, indicating a fast reaction of ROS with PUFAs, contributing to lipid peroxidation and ferroptosis. No alterations in ROS or lipid peroxide levels were observed in MDA‐MB‐231 exposed to PURE_G4_‐OCEI_24_ (Fig. 3D). Cells are continuously exposed to oxidative damage but only undergo oxidative stress when an imbalance arises due to increased ROS production or a reduced ability to scavenge them [69].

Assuming that in ferroptosis ROS scavenging is compromised leading to the accumulation of lipid peroxides [70, 71], the expression of genes encoding enzymes involved in cysteine and glutathione bioavailability and ROS scavenging showed a significant impact of PURE_G4_‐OCEI_24_ in HCC1806 (Fig. 3E). Despite no differences in GPX4, which is responsible for scavenging lipid peroxides and converting them into nontoxic lipid alcohols [72], the expression of CGL and CTH was increased in cells exposed to PURE_G4_‐OCEI_24_. CGL encodes the limiting enzyme in glutathione synthesis, and CTH encodes the CSE enzyme important in cysteine synthesis and degradation [73, 74]. These results indicate that cells are experiencing oxidative stress and undergoing metabolic remodeling by activating the expression of genes that will restore the redox balance, although ferroptosis is triggered and may contribute to cell death in cells exposed to PURE_G4_‐OCEI_24_. The production of hydrogen sulfide (H_2_S) from cysteine degradation may be an attempt to avoid ferroptosis [75]. xCT, the main transporter of cyst(e)ine used by cancer cells [76, 77, 78], is considered an inhibitor of ferroptosis, and cancer cells undergoing this cell death process present decreased levels of xCT [77, 79, 80]. The activation of ferroptosis by PURE_G4_‐OCEI_24_ in HCC1806 is thus suggested by the decreased levels of xCT (Fig. 3F), even with no alterations in ROS and lipid peroxide levels at this timepoint (Fig. 3D).

The anticancer potential of both PURE_G4_‐OEI_48_ and PURE_G4_‐OCEI_24_ was validated in the HCC1806 xenograft tumors murine model (Fig. 4), with treated mice presenting decreased tumor volumes (Fig. 4A,C). The LD content was altered in tumors treated with PURE_G4_‐OEI_48_ and PURE_G4_‐OCEI_24_, showing an opposite correspondence in the levels of lipid peroxides in tumors treated with PURE_G4_‐OEI_48_ and PURE_G4_‐OCEI_24_ (Fig 5A,B). These findings show an interplay between LD content and peroxidation and agree with previous descriptions that TNBC cells are susceptible to ferroptosis [81]. LDs originate from the endoplasmic reticulum and function as storage organelles for neutral lipids, primarily triacylglycerol and sterol esters [82]. As a result, LDs regulate lipid uptake, storage, and utilization based on cellular needs. Although the precise mechanism by which neutral lipids accumulate and form LDs remains unclear [83], this process is commonly observed in cancer. LDs play a protective role by shielding cells from ROS toxicity and serve as energy reserves, thereby promoting cell survival, aggressiveness, and resistance to therapy [84]. Therefore, the alteration in LD content in tumors treated with dendrimers reinforces the fact that PURE_G4_‐OEI_48_ and PURE_G4_‐OCEI_24_ interfere with the redox balance of HCC1806‐derived tumors.

The oxidative stress was further confirmed by measuring the xCT and Nrf2 levels (Fig 5C,D). The levels of xCT, stated as an inhibitor of ferroptosis, tended to increase but not significantly in tumors from mice treated with PURE_G4_‐OEI_48_ compared with the control untreated tumors, while no changes were observed in tumors from mice treated with PURE_G4_‐OCEI_24_. The tendency to increase xCT expression upon PURE_G4_‐OEI_48_ exposure suggests, in a scenario of long exposure, that oxidative stress may activate xCT expression as a cellular detoxification response [37]. Accordingly, the Nrf2 levels were significantly increased in tumors from mice treated with PURE_G4_‐OEI_48_ compared with the control untreated tumors (Fig 5C,D). Nrf2 is a transcription factor that regulates xCT expression, and both will account for oxidative stress control, for instance, through activation of glutathione production [37, 85], since Nrf2 is the master controller of redox responses [36]. Furthermore, HCC1806‐derived tumors treated with PURE_G4_‐OEI_48_ and PURE_G4_‐OCEI_24_ exhibited increased levels of ATG12, an autophagy‐related protein [86], compared with the control group (Fig 5E,F). However, only the PURE_G4_‐OCEI_24_ treatment induced a significantly higher expression of ATG12 compared with tumors from untreated mice. PURE_G4_‐OCEI_24_ induced stress may activate autophagy as a survival mechanism, which aligns with the absence of significant differences in oxidative stress markers, such as lipid peroxides accumulation, and xCT and Nrf2 expression in tumors from mice treated with this dendrimer.

*In vivo* studies investigating dendrimer toxicity remain limited, with most research focusing primarily on poly(amidoamine) (PAMAM) dendrimers. Systemically, dendrimers, especially cationic dendrimers, have been linked to hemolytic activity and the formation of blood clots [87, 88]. For instance, poly(propylene imine) (PPI) dendrimers have been reported to induce autophagy in the nervous system, leading to adverse effects, such as ataxia. Other studies have shown that PAMAM dendrimers can accumulate in organs, such as the heart, liver, kidneys, and lungs following oral administration [89, 90, 91].

However, to date, no studies have evaluated the systemic toxicity of PURE dendrimers, likely due to their recently uncovered anticancer potential. Our findings on intratumoral administration suggest promising outcomes despite certain limitations. Unlike conventional PAMAM dendrimers, which have terminal surface amines known to contribute to toxicity [91], PURE dendrimers do not expose terminal amine groups, potentially reducing their cytotoxic effects. Additionally, dendrimer‐associated toxicity is often correlated with higher‐generation structures. Polyurea dendrimers, while not inherently biodegradable, pose some challenges for clinical application. Nevertheless, the POXylated intermediate PURE_G4_‐OEtOx_48_ exhibits a hydrodynamic radius of approximately 4 nm, which is small enough to allow efficient renal clearance. Its cationic surface charge further enhances elimination through the kidneys [92], helping to address concerns regarding nondegradable urea monomers. However, we observed that PURE_G4_‐OEI_48_ and PURE_G4_‐OCEI_24_ present a size over 24 nm, which may imply a more specific mechanism for clearance; hence, more studies are needed, including *in vivo* studies to address bioavailability and detoxification of these dendrimers.

Regarding the stability of the dendrimers, our previous assays showed that PURE_G4_‐OCEI_24_ (EC_50_ concentration) incubated with FBS and culture media for 72 h is stable as the characteristic peaks were detected by ^1^H‐NMR spectroscopy. Still, in the case of PURE_G4_‐OEI_48_ under the same incubation conditions, no peaks were found, which may indicate degradation. Nevertheless, it should be stressed that cellular uptake is fast, thus precluding degradation before internalization. Despite these considerations, preliminary results are highly encouraging. No signs of toxicity were observed in major organs, such as the heart, liver, or kidneys (Fig. 4D,E).

Both PURE_G4_‐OEI_48_ and PURE_G4_‐OCEI_24_ have shown potential as modulators of oxidative stress in TNBC models, both *in vitro* and *in vivo*. However, their redox‐disrupting mechanism of action raises concerns about possible effects on healthy cells. In particular, excessive ROS production could lead to oxidative damage in normal tissues, including DNA damage and lipid peroxidation, similar to effects observed in TNBC‐derived tumors. In a systemic context in mice, PURE_G4_‐OEI_48_ showed no toxic effects based on the toxicity serum markers and the histological analysis. PURE_G4_‐OCEI_24_ caused no morphological alterations; however, increased levels of aspartate aminotransferase indicate hepatic toxicity [93, 94] (Fig 4D,E). The tissue sections (Fig. 4E) show that the histological morphology of the organs typically associated with toxicity—heart, liver, and kidneys—is preserved, with no signs of inflammation, necrosis, or fibrosis, which would indicate cellular injury [95, 96] due to dendrimer application. The absence of histological alterations supports the conclusion that, at least in topical application, the tested dendrimers do not induce significant systemic toxicity. Nevertheless, the increased levels of aspartate aminotransferase may indicate that PURE_G4_‐OCEI_24_ detoxification happens in the liver and proves that oxidative stress generation is a mechanism of action of PURE_G4_‐OCEI_24_, as observed *in vitro* in HCC1806 cells. No evidence of nephrotoxicity, which affects mainly renal tubular cells [97], was observed for both dendrimers, since the cell death levels of the HK‐2 exposed to dendrimers were not altered (Fig. 2F), as well as the creatinine levels in the blood serum of treated mice (Fig. 4D). These results align with the fact that the liver is the main organ controlling the redox state of the organism and oxidative stress is a principal cause of hepatic diseases due to toxicity [98, 99].

Overall, this study demonstrates that PURE_G4_‐OEI_48_ and PURE_G4_‐OCEI_24_ may be a therapeutic alternative to treat TNBC, inducing apoptosis and necroptosis together with redox imbalance and ferroptosis activation as the main mechanisms of action. However, considering the few results on blood coagulation and hemolysis (Fig 5G–I), PURE_G4_‐OEI_48_ seems to be the best option. Nevertheless, more studies are needed to fully characterize the anticancer efficacy and safety of this dendrimer as a drug.

Moreover, these nanoparticles offer the potential for designing combined therapeutic approaches by loading dendrimers with cytotoxic drugs. This allows us to leverage the chemical properties of the dendrimers, which can interact more specifically with cancer cell membranes due to their composition and charge, while also serving as efficient vehicles for drug delivery.

## Conflict of interest

The authors declare no conflict of interest.

## Author contributions

AC wrote the first draft and contributed to the conceptualization, experiments, methodology, and data validation, and discussed and revised the final version. BA and CM contributed to the experiments, methodology, and data validation, and discussed and revised the final version. CF‐D, FG, FS, and JR contributed to the methodology, and discussed and revised the final version. SA contributed to the methodology and supervision, and discussed and revised the final version. VDBB contributed to the conceptualization, methodology, and funding acquisition, and discussed and revised the final version. JS wrote the first draft, contributed to the conceptualization, data validation, and funding acquisition, and discussed and revised the final version.

## Supporting information


**Fig. S1.** ^1^H NMR spectrum of PURE_G4_‐OCEI_24_.

**Fig. S2.** Cell death positive controls with 10% DMSO (v/v) and 50 ng·mL^−1^ of TNFα.

## Data Availability

All data supporting the findings of this study are included in this manuscript. The raw data will be made available upon request.
