# Supplementary figures and images for: Targeting TNBC: core–shell polycationic polyurea dendrimers with inherent anticancer activity
Source: FEBS Open Bio. 2026 Mar 16;16(5):944–65. doi: 10.1002/2211-5463.70144 (PMC13145356; doi:10.1002/2211-5463.70144)

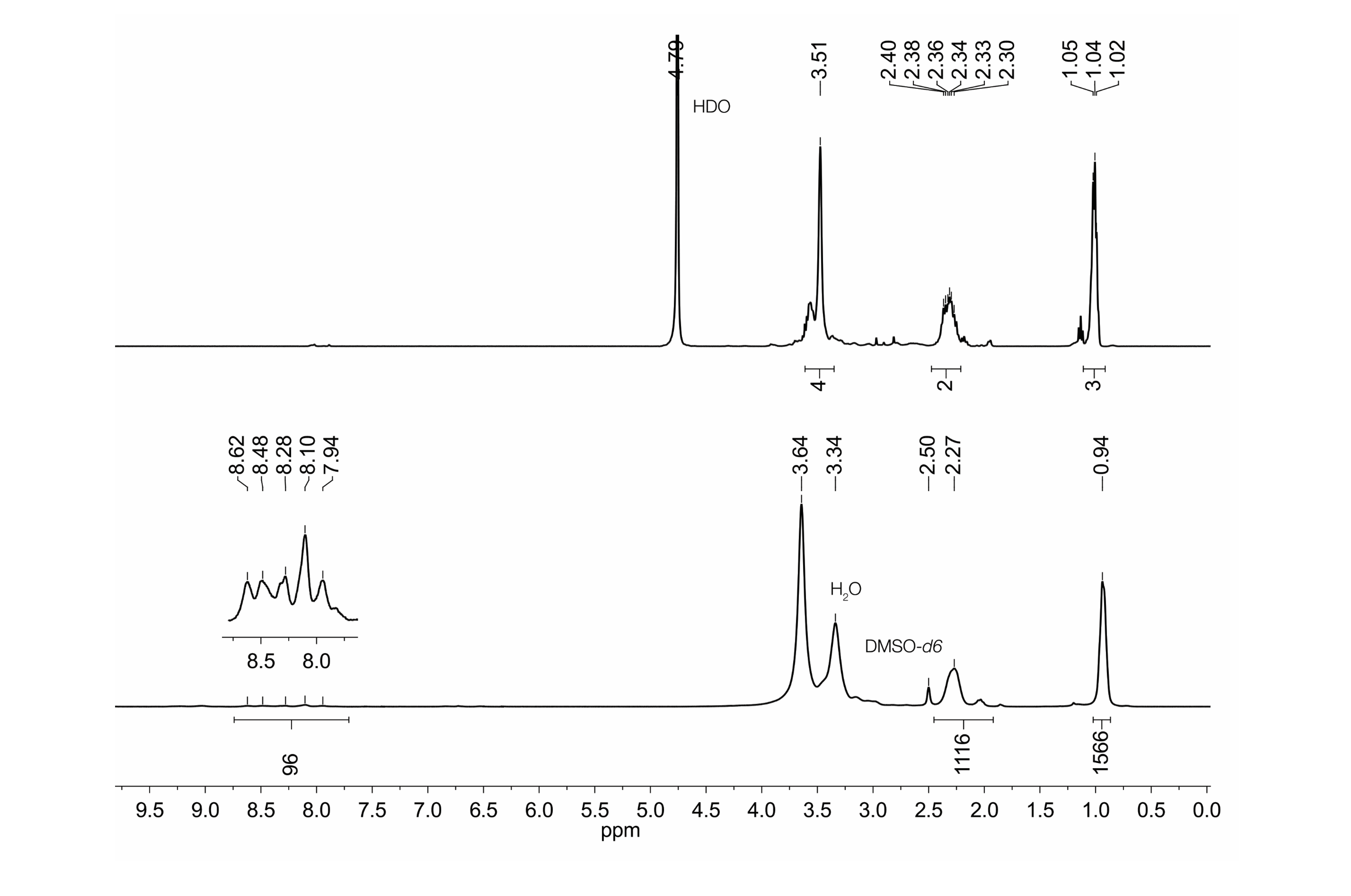

Supplement: Supplementary file 1 — Fig. S1. 1H NMR spectrum of PUREG4‐OCEI24. [file FEB4-16-944-s002.tif]

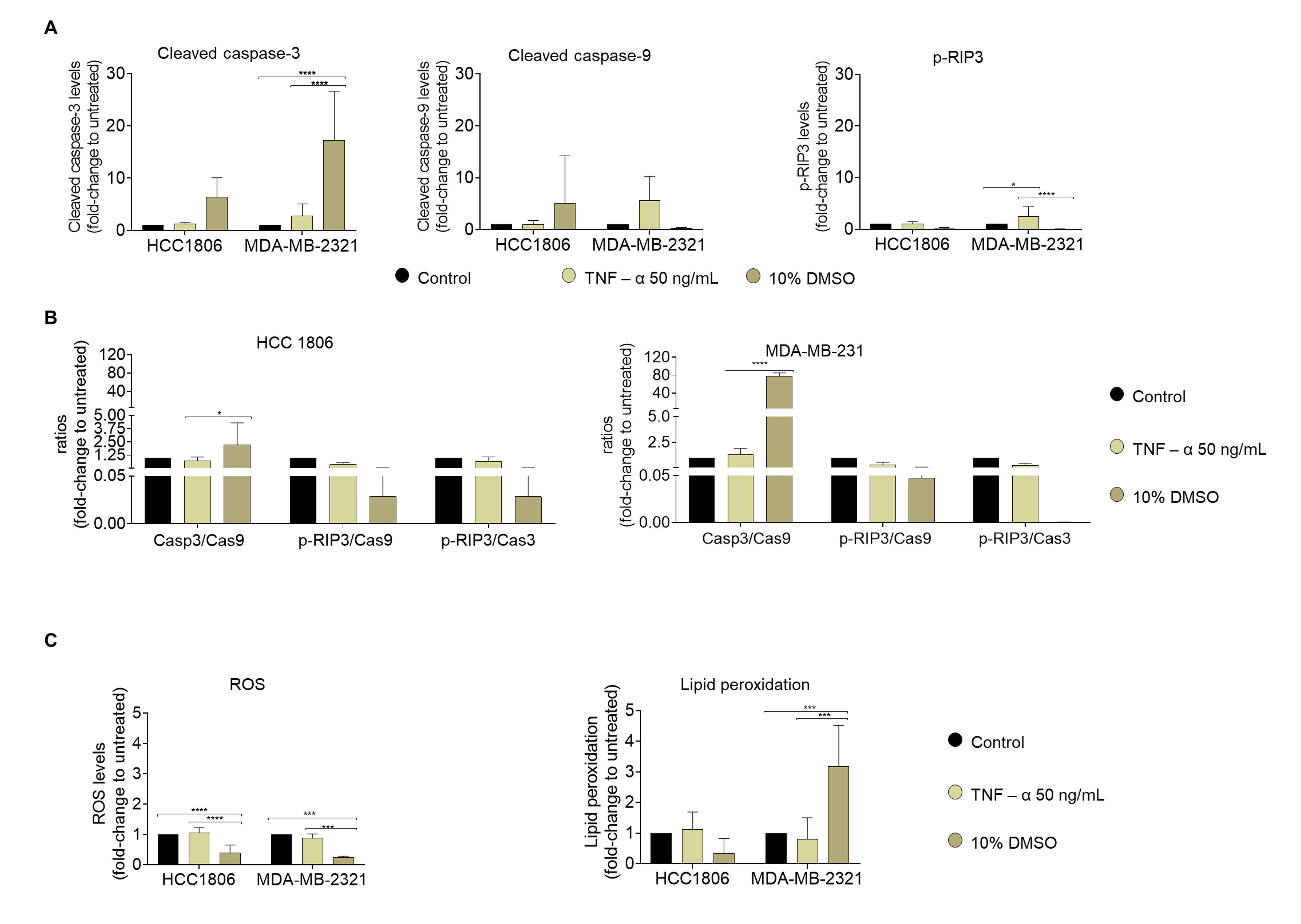

Supplement: Supplementary file 2 — Fig. S2. Cell death positive controls with 10% DMSO (v/v) and 50 ng·mL−1 of TNFα. [file FEB4-16-944-s001.tif]
